# Numerical simulation and optimization of BaZrSe_3_/ZnS heterojunction solar cells: achieving high performance

**DOI:** 10.1039/d5ra05711f

**Published:** 2025-10-21

**Authors:** Elsammani Ali Shokralla, Arslan Ashfaq, Ubaid Ur Rehman, Hind Albalawi, Zahra Bayhan, Sarah A. Alsalhi, Shoug M. Alghamdi, M. Musa Saad H.-E.

**Affiliations:** a Department of Physics, Faculty of Science, Al-Baha University Alaqiq 65779-7738 Saudi Arabia; b Department of Physics, Emerson University Multan Multan 60000 Pakistan arslan.ashfaq201@gmail.com arslan.ashfaq@eum.edu.pk; c School of Physics, State Key Laboratory of Crystal Materials, Shandong University Jinan Shandong 250100 China; d Department of Physics, College of Sciences, Princess Nourah bint Abdulrahman University (PNU) P. O. Box 84428 Riyadh 11671 Saudi Arabia; e Department of Physics, College of Science, Taibah University Yanbu Governorate Saudi Arabia; f Department of Physics, College of Science, Qassim University Buridah 51452 Saudi Arabia

## Abstract

In this work, a ZnO:Al/ZnO/ZnS/BaZrSe_3_/Au heterojunction solar cell was numerically investigated using SCAPS-1D to optimize its structural and electronic parameters for high photovoltaic performance. The effects of absorber thickness (50 nm–6.0 μm), buffer layer thickness (10–100 nm), doping densities, defect states, operating temperature, and back metal contacts were systematically studied. The results revealed that increasing the BaZrSe_3_ absorber thickness enhanced the short-circuit current density (*J*_sc_) due to improved light absorption, with an optimum thickness of 2.0 μm balancing carrier generation and recombination. The ZnS buffer layer exhibited optimum performance at 20 nm, ensuring efficient charge transfer without increasing resistive losses. The acceptor doping concentration in BaZrSe_3_ strongly influenced the device properties, with *N*_A_ = 10^18^ cm^−3^ yielding the maximum PCE of 22.77%. Similarly, an optimized donor doping density of 10^19^ cm^−3^ in the buffer enhanced carrier extraction. Defect density analysis showed that PCE remained stable up to *N*_T_ = 10^14^ cm^−3^, beyond which recombination dominated, reducing efficiency. Temperature-dependent simulations indicated a decline in PCE from 22.92% at 300 K to 17.87% at 360 K due to enhanced carrier recombination. Finally, the choice of back contact significantly affected performance, with a high work-function metal (5.9 eV) achieving superior results, including PCE = 29.46%, *V*_oc_ = 0.7528 V, *J*_sc_ = 46.38 mA cm^−2^, and FF = 84.37%. These results highlight the promising potential of BaZrSe_3_ as a lead free absorber material for next-generation thin film solar cells, where optimization of thickness, doping, and contact engineering play a crucial role in maximizing device efficiency.

## Abstract 2

SCAPS-1D simulations of ZnO:Al/ZnO/ZnS/BaZrSe_3_/Au solar cells show optimal absorber thickness (2.0 μm), buffer (20 nm), doping, and contacts, yielding 29.46% PCE. BaZrSe_3_ emerges as a promising lead-free absorber.

## Graphical abstract

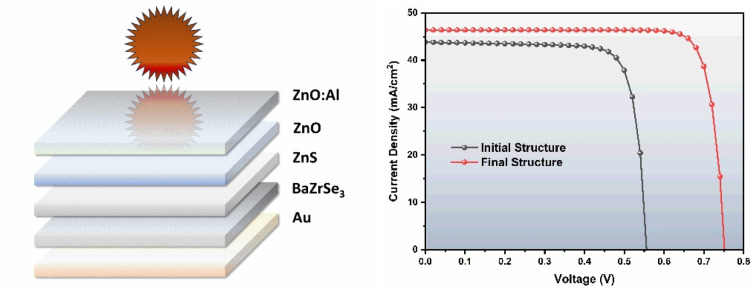

## Introduction

Research on solar energy and the development of solar cell technologies has attracted widespread interest since Alexandre Edmond Becquerel first explored the concept in 1839.^1^ Over the years, the field has progressed considerably, driven by the growing demand to harness the sun's abundant and renewable energy for practical applications. Becquerel's pioneering work initiated the study of photovoltaic (PV) phenomena, setting the stage for a scientific journey that has become essential to global efforts toward clean and sustainable energy solutions. In the observation, when light struck a metal back electrode occupied in an electrolyte result, a small electric current was generated. This discovery resulted in the invention of the PV outcome, which helps as the foundation for modern solar cell technology. Building on this pioneering discovery, substantial research efforts have been made to examine the unique features of many materials, with the aim of improving PCE.^2^

Solar technology has developed over time, with each generation introducing distinct challenges and materials. The first generation primarily utilized monocrystalline and multicrystalline silicon (m-Si) architectures. Although silicon-based devices dominated the commercial market, their wider adoption was hampered by high production costs.^3^ To address these issues, the second generation of cells arose, using thin film technologies derived from materials such as CdTe, CIGS, and amorphous silicon.^4,5^ These advances aimed to reduce production costs and increase performance, but also created new technological challenges. Despite these hurdles, solar cell research took a new turn, opening the path for the 3rd generation. This generation includes breakthroughs such as organic–inorganic hybrid halide structures, which are prized for their high efficiency, low production costs, and good opto-electronic properties, become the promising candidates for photovoltaic applications. Despite reaching an impressive PCE of 26.10% according to the National Renewable Energy Laboratory (NREL) records, their widespread adoption remains limited due to the uncertainty of organic components and concerns over Pb toxicity.^6^ Lead is a highly toxic element, with its use strictly regulated or banned in many nation states due to its injurious effects on environment and human health.^7^ The risk increases when lead volatilizes at high temperatures during procedures like sintering and calcination, releasing harmful pollutants into the atmosphere.^8^ This pollution was led to lead poisoning, causing symptoms such as muscle pain, fatigue, and abdominal discomfort, while posing serious risks to brain and neurological development. As a result, there is a significant effort to create naturally stable, Pb-free materials, including chalcogenide perovskites, as safer, sustainable alternatives.^9^

The perovskite structure is ABX_3_, with A is cations with consisting of group II, B is the transition metals consisting of group IV, and X referring to chalcogen anions. These materials are highly promising due to their eco friendly and non toxic properties.^10,11^ These compounds possess a distorted perovskite framework and exhibit semiconducting properties, with band gaps spanning from 0.3 to 2.3 eV. They possess direct energy bandgaps and maximum absorption spectrum, similar to conventional opto-electronic semiconductors such as GaAs. Furthermore, hafnium (Hf) and zirconium (Zr) compounds exhibit significant band dispersion, indicating high carrier mobility.^12^ These qualities make them ideal for photovoltaic, like as the SrZrS_3_, BaZrS_3_, and BaZrSe_3_, all of which have bandgaps suitable for solar applications.^2^

BaZrS_3_ is unique among chalcogenide perovskites, attracting great research interest because to its exceptional eco-friendly stability, lead free composition, superior absorption, and impressive carrier movement. The absorption co-efficient (*α*) is higher than that of several standard solar cell absorbers, showing considerable potential for effective photo-generated charge carrier collection. According to research, the ideal bandgap for single junction solar cells is approximately 1.35 eV. However, BaZrS_3_ typically has a bandgap of 1.70 to 1.90 eV, which is slightly higher than the optimum range. To address this challenge, scientists have shifted their attention to BaZrSe_3_, which features energy band gap range from 1.0–1.45 eV closer to the ideal range for efficient solar cell performance. Ong *et al.*'s calculations revealed especially lower band-gap of 1.10 eV for BaZrSe_3_ highlighting its potential as an effective material for solar cell light absorption.^13^

In the field of chalcogenide perovskite, extensive research has focused on unraveling the intricate relationships between the various layers of the cell. The performance and reliability of chalcogenide PSCs heavily rely on the seamless integration of these layers, as each plays a crucial role in influencing the device overall functionality and performance. Improving device stability and performance hinges on the heterostructure interface between the absorber layer and the ETL, which plays a vital role in ensuring efficient charge transport and minimizing energy losses.^14,15^

ZnS emerges as a promising candidate for the buffer layer due to its exceptional properties, making it an attractive option for chalcogenide heterojunction. With its excellent transparency in the high electron mobility, maximum visible range, and wide energy band gap of around 3.68 eV, and robust thermal and electro-chemical stable, ZnS proves to be a highly suitable choice for buffer layer. Its cost-effectiveness, combined with advantageous opto-electronic properties, further highlights ZnS suitability for this critical role.^16^

In the current work, SCAPS-1D numerical software using to investigate the potential of absorber layer BaZrSe_3_ in the heterojunction ZnO:Al/ZnO/ZnS/BaZrSe_3_/Au structure. This study seeks to improve the understanding of selenium-based heterojunction optimization by thoroughly adjusting key parameters like thickness, acceptor and donor doping density, defect density and temperature. The reduction of charge carrier recombination with defect density concentration were also optimized. The study purposes to offer valuable insights to address challenges related to efficiency and stability in heterojunction technology.

### Numerical simulation and device structure

The SCAPS-1D program (version 3.3.12) was used to simulate and analyze the behavior of solar cell structures. The software, developed by the Department of Electronics and Information Systems at Ghent University, is a widely recognized one-dimensional tool for thin-film device modeling.^17^ Its operation is based on the electrical and optical characteristics of the chosen semiconductor materials, allowing reliable predictions of device performance. SCAPS-1D was particular for this study because of its proven accuracy in handling complex heterojunctions and its capacity to provide detailed insights into a variety of material systems.

A key advantage of SCAPS-1D lies in its user-friendly interface and robust defect modeling framework, which allows researchers to examine the influence of numerous intrinsic and extrinsic parameters. This versatility makes it particularly suitable for studying advanced chalcogenide-based heterojunction devices aimed at sustainable energy applications. The software was simulated essential processes such as band alignment, charge generation, recombination mechanisms, and defect distributions, thereby providing a comprehensive picture of device physics. Furthermore, it numerically solves Poisson's equation together with the electron and hole continuity equations, enabling precise evaluation of critical photovoltaic parameters.1
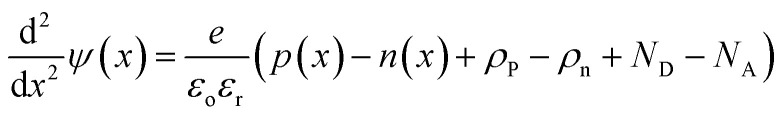
2
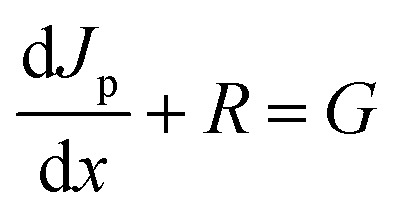
3
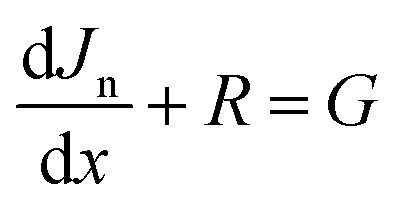


The physical constraints are expressed as follows: *e* is the elementary charge, *Ψ* denotes the electrostatic potential, *ε*_0_ and *ε*_r_ is the vacuum and relative permittivity, respectively. The carrier concentrations are given by n (electrons) and p (holes), while their spatial charge densities are described as *ρ*_n_ and *ρ*_p_. The terms *J*_n_ and *J*_p_ indicate the current densities associated with electrons and holes, respectively. Furthermore, *R* refers to the recombination rate, and *G* designates the carrier generation rate.

**Fig. 1:**
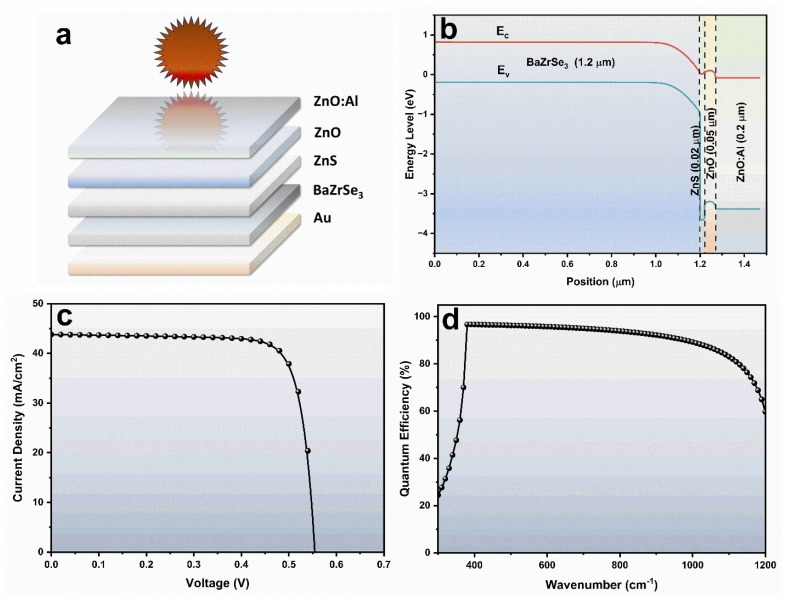
(a) Schematic of the solar cell structure, (b) corresponding energy band diagram, (c) current density–voltage characteristics (d) quantum efficiency spectrum of the baseline device configuration.

In this configuration, Fig. 1 presents (a) the solar cell structure ZnO:Al/ZnO/ZnS/BaZrSe_3_/Au (b) the corresponding energy band diagram of the structure. ZnO:Al is employed as the front contact, while ZnO is served as the window layer, which ensuring high optical transmission with maximum protection of the devices. The ZnS layer functions as the buffer, acting as n-type material with high electron mobility. It minimizes interfacial charge accumulation and facilitates charge transport, thereby improving overall device performance. The BaZrSe_3_ layer is utilized as the primary light absorber, and gold (Au) is applied as the back electrode to complete the cell structure.

The simulation process was initiated using the input parameters summarized in Table 1. These parameters were carefully chosen on the base of previous reports to guarantee accuracy and reliability. For consistency and direct comparability, all numerical simulations were carried out at a fixed room temperature under standard AM 1.5G illumination, corresponding to a light intensity of 1000 W m^−2^.

**Table 1:** Initial conditions and material parameters for heterojunction solar cell simulations in SCAPS-1D

Properties	BaZrSe_3_	ZnS	ZnO	ZnO:Al
Thickness (nm)	1200	20	50	200
*E*_g_ (eV)	1.01	3.68	3.3	3.3
*N*_C_ (cm^−3^)	2.2 ×10^18^	1.5 ×10^18^	4.1 ×10^18^	4.1 ×10^18^
*N*_v_ (cm^−3^)	1.8 × 10^19^	1.8 × 10^19^	8.2 × 10^18^	8.2 × 10^18^
*χ* (eV)	4.5	4.5	4.55	4.53
*ε* (eV)	4.19	8.32	8.0	8.0
*N*_A_ (cm^−3^)	10^16^	0	0	0
*N*_D_ (cm^−3^)	0	10^18^	10^10^	10^10^
*N*_T_ (cm^−3^)	10^14^	10^14^	10^14^	10^14^
*μ*_p_ (cm^2^ V^−1^ s^−1^)	113	25	20	20
*μ*_h_ (cm^2^ V^−1^ s^−1^)	58	100	100	100
References	18	19	19	19

Fig. 1(c) presents the quantum efficiency (QE), while Fig. 1(d) illustrates the current density–voltage (*J*–*V*) characteristics of the initial and proposed device structures. For the initial configuration, the simulated performance parameters were obtained as: open-circuit voltage (*V*_oc_) = 0.5568 V, short-circuit current density (*J*_sc_) = 43.81 mA cm^−2^, fill factor (FF) = 79.77%, and power conversion efficiency (PCE) = 19.46%.

## Results and discussion

### Effect of thickness on absorber and buffer performance

The efficiency of a heterojunction solar cell is strongly effect by the selection of the absorber layer and its thickness. To evaluate this effect, the BaZrSe_3_ absorber thickness was varied between 50 nm to 6.0 μm, while the thicknesses of the remaining layers were kept constant.

**Fig. 2:**
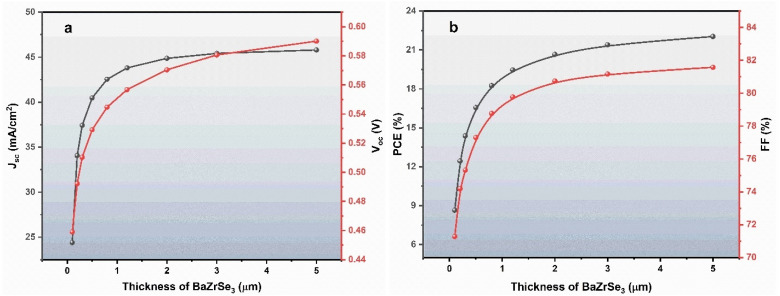
Effect of BaZrSe_3_ absorber thickness on heterojunction performance (a) *J*_sc_ & *V*_oc_ (b) FF & PCE .

Fig. 2(a and b) demonstrates how variations in the BaZrSe_3_ thickness affect the photovoltaic properties of the ZnO:Al/ZnO/ZnS/BaZrSe_3_/Au heterostructure. Increasing the perovskite thickness from 50 nm to 6.0 μm leads to a steady enhancement in key performance indicators, including *J*_sc_, *V*_oc_, FF, and PCE. At lower thicknesses, the rapid improvement in performance is primarily associated with stronger photon absorption region and more effective carrier generation.

The open-circuit voltage (*V*_oc_) rises quickly with thickness up to ∼1000 nm, stabilizing around 0.55 V thereafter. This behavior indicates that charge extraction improves initially as recombination losses are minimized, but beyond this point *V*_oc_ saturates since the gain in photogenerated carriers is counterbalanced by increased recombination within the bulk of the absorber. This trend was described using the diode model equations,^20^4
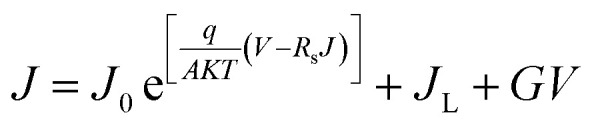
5
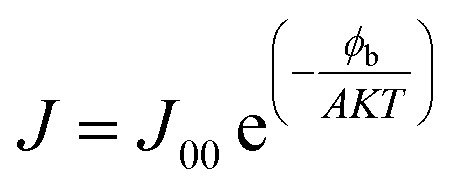
6
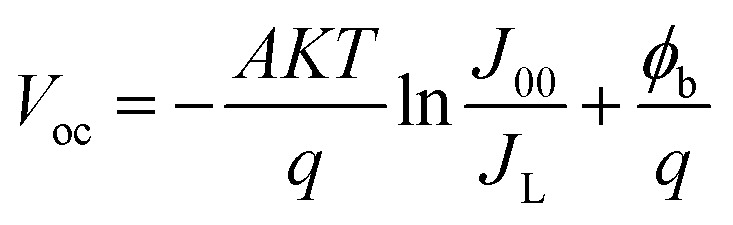
Here, *J* represents the total current–density, *J*_L_ the photo generated current, *J*_0_ the saturation current, and *J*_00_ a pre-exponential factor. The parameters *q*, *A*, and *T* denote the elementary charge, ideality factor, and absolute temperature, respectively, while *R*_s_ and *G* refer to the series resistance and shunt conductance, and *ϕ*_b_ corresponds to the barrier height.

The *J*_sc_ also exhibits a thickness-dependent trend. At very thin layers (50–200 nm), incomplete absorption of longer-wavelength photons reduces carrier generation, keeping *J*_sc_ relatively low. The enhance of the thickness are caused to more photons absorber, resulting in higher carrier generation and improved *J*_sc_ values. However, once the absorber becomes thicker than the effective diffusion length, carrier recombination increases, causing *J*_sc_ to saturate rather than increase indefinitely. This explains why performance growth is more substantial below ∼1.5 μm but less pronounced at higher thicknesses.

The overall device efficiency (PCE) follows a similar pattern: low efficiency at minimal thickness due to poor absorption, significant enhancement at moderate thicknesses owing to balanced light absorption and carrier collection, and eventual saturation at larger thicknesses due to recombination and resistive losses.^21^ The FF also improves with absorber thickness as recombination at the junction is suppressed, but its growth rate diminishes once the device reaches optimal thickness.

Although simulations show continued improvement up to 6.0 μm, such high thicknesses are not practical in experimental fabrication because of excessive material consumption, long deposition times, and challenges in maintaining high crystallinity. Additionally, carriers generated deep inside a very thick absorber often fail to reach the junction, reducing their contribution to the photocurrent. Considering these trade-offs, a moderate BaZrSe_3_ thickness of ∼2.0 μm is identified as the most suitable value. This thickness ensures efficient optical absorption, minimizes recombination, and balances device performance with realistic fabrication constraints.

**Fig. 3:**
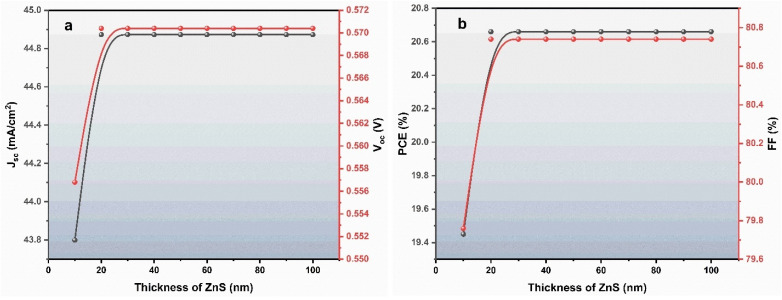
Variation in device performance (a) *J*_sc_ & *V*_oc_ (b) FF & PCE with ZnS buffer layer thickness.

Fig. 3(a and b) shows the effect of ZnS buffer layer thickness on the photovoltaic performance of the device. The thickness of the ZnS layer was varying from 10 nm to 100 nm. It was observed that increasing the buffer thickness from 10 nm to 20 nm leads to noticeable improvements in *V*_oc_, *J*_sc_, FF, and PCE. Beyond 20 nm, however, the device performance remains nearly constant, indicating that further increases in ZnS thickness do not provide additional benefits.

The improvement at 20 nm was attributed to the effective role of ZnS in reducing interface recombination between the ZnO window and the BaZrSe_3_ absorber. At 10 nm, the coverage of the buffer layer is insufficient to fully passivate interface defects, which results in increased carrier recombination and lower performance. By increasing the thickness to 20 nm, the ZnS layer becomes continuous and uniform, effectively blocking recombination pathways and facilitating improved carrier separation. This results in higher *J*_sc_ due to better charge collection, as well as improved *V*_oc_ and FF.

Once the ZnS thickness exceeds 20 nm, the parameters saturate because the layer has already reached its optimal passivation effect. Additional thickness only introduces unnecessary series resistance and does not enhance light absorption, since ZnS is a wide bandgap material (3.68 eV) that does not contribute to photocurrent generation. As a result, PCE does not increase further for thicker ZnS layers.

Based on these observations, a ZnS thickness of 20 nm was selected as the optimum value. This thickness provides sufficient interface passivation and stability while avoiding excess material usage and resistive losses.

### Effect of acceptor density of BaZrSe_3_

**Fig. 4:**
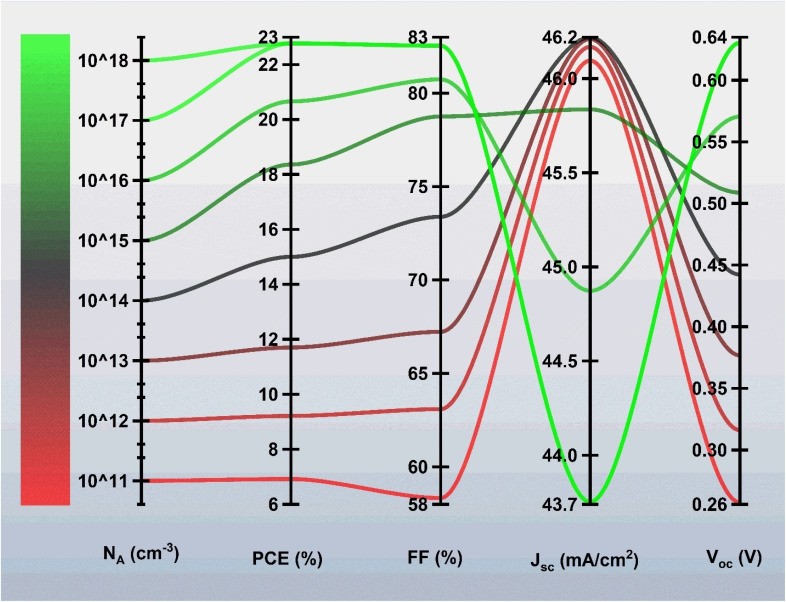
Variation of solar cell characteristics with absorber acceptor doping content.

Fig. 4 illustrates the impact of changing the acceptor doping density (*N*_A_) of the BaZrSe_3_ absorber layer on the photovoltaic parameters of the ZnO:Al/ZnO/ZnS/BaZrSe_3_/Au device. The doping content was tuned from 1 × 10^11^ to 1 × 10^18^ cm^−3^. At very low doping density (*N*_A_ = 1 × 10^11^ cm^−3^), the device exhibits a high *J*_sc_ ≈ 46.09 mA cm^−2^ but a low *V*_oc_ ≈ 0.2575 V. This occurs because the weakly doped absorber forms a shallow built-in electric field, which enhances carrier diffusion and collection but fails to provide sufficient junction potential, thereby limiting *V*_oc_.

As the *N*_A_ increases, the depletion region becomes narrower, and the built-in potential rises, which improves charge separation and reduces recombination. At *N*_A_ = 1 × 10^18^ cm^−3^, the device reaches a *V*_oc_ of 0.6304 V and an optimized *J*_sc_ of 43.05 mA cm^−2^. Although *J*_sc_ decreases slightly due to the reduced depletion width and higher recombination at elevated doping levels, the gain in *V*_oc_ and FF compensates for this reduction. The maximum power conversion efficiency (PCE = 22.77%) and FF (82.54%) are obtained at this doping concentration.

This behavior reflects the trade-off between photocurrent and photovoltage as governed by doping density. Lower *N*_A_ favors current generation but suppresses voltage due to insufficient barrier height, while higher *N*_A_ strengthens the junction, boosting *V*_oc_ and FF at the expense of some current. The optimum doping level of 1 × 10^18^ cm^−3^ ensures a balanced performance, yielding the highest efficiency by maintaining reasonable *J*_sc_ while maximizing *V*_oc_ and FF. Therefore, *N*_A_ = 1 × 10^18^ cm^−3^ was selected as the optimal doping concentration for the BaZrSe_3_ absorber in this device structure.

### Impact of donor density of ZnS

**Fig. 5:**
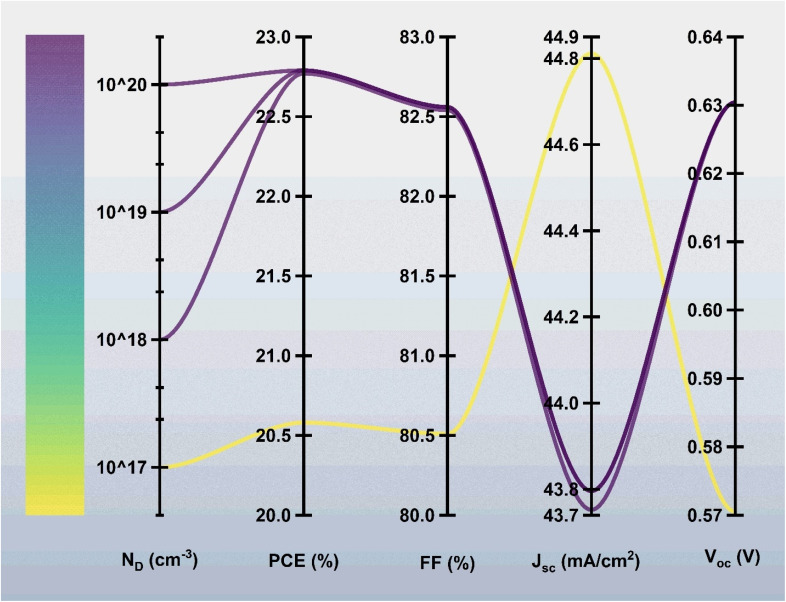
Effect of ZnS donor carrier concentration on the device characteristics.

Fig. 5 shows the influence of ZnS donor doping concentration (*N*_D_) on the photovoltaic performance of the ZnO:Al/ZnO/ZnS/BaZrSe_3_/Au solar cell. The doping density was varied from 1 × 10^16^ to 1 × 10^20^ cm^−3^. The results reveal a steady increase in device efficiency with higher donor concentration, with PCE improving from 20.58% at *N*_D_ = 1 × 10^16^ cm^−3^ to 22.79% at *N*_D_ = 1 × 10^20^ cm^−3^.

At lower donor concentrations, the ZnS buffer exhibits limited conductivity, which restricts electron transport and increases series resistance, thereby reducing overall device performance. As *N*_D_ increases, the electrical conductivity of the buffer layer improves, minimizing resistive losses and enhancing carrier collection across the heterojunction. This results in better current extraction, higher *V*_oc_, and improved FF.

However, excessively high donor doping was introducing drawbacks such as increased interface recombination, tunneling effects, or potential band misalignment at the ZnS/BaZrSe_3_ interface. To balance these factors, an optimized doping density of 1 × 10^19^ cm^−3^ was selected. At this concentration, the device benefits from reduced resistive losses and efficient carrier transport, while avoiding the detrimental effects that may arise at extremely high doping levels.

Therefore, *N*_D_ = 1 × 10^19^ cm^−3^ for the ZnS buffer is identified as the optimal choice, providing a stable efficiency of ∼22.79% with reliable device operation.

### Impact of defect density of the BaZrSe_3_ layer

**Fig. 6:**
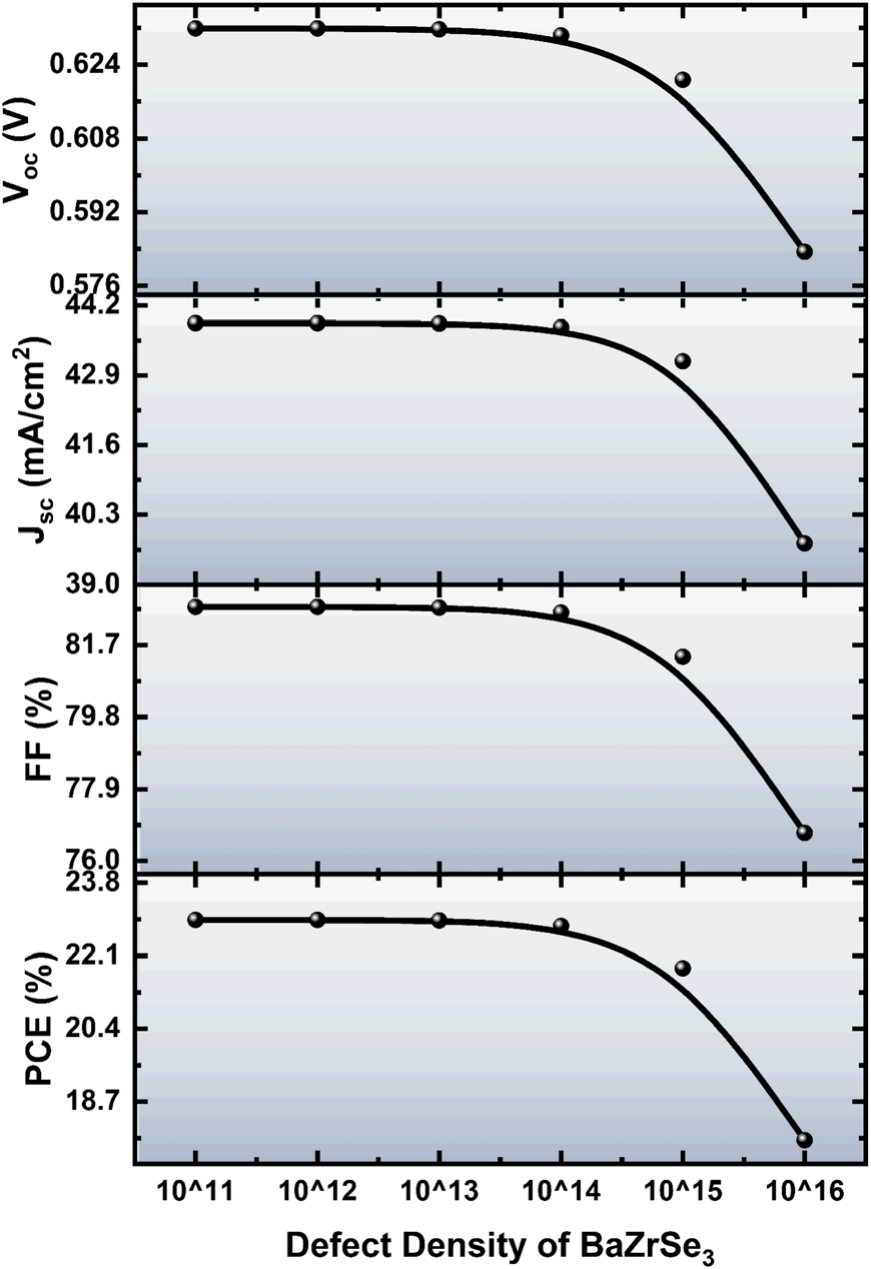
Impact of defect density (*N*_T_) in the BaZrSe_3_ layer on device performance.

Fig. 6 illustrates the effect of defect density (*N*_T_) in the BaZrSe_3_ absorber layer on the photovoltaic characteristics of the ZnO:Al/ZnO/ZnS/BaZrSe_3_/Au solar cell. The *N*_T_ value was varied from 1 × 10^11^ to 1 × 10^16^ cm^−3^. The simulation results show that for defect densities up to 1 × 10^14^ cm^−3^, the device maintains stable performance, with *J*_sc_, *V*_oc_, FF, and PCE remaining nearly constant. This indicates that at low *N*_T_, the density of trap states is insufficient to significantly affect charge carrier transport or recombination.

Beyond *N*_T_ = 1 × 10^14^ cm^−3^, all performance parameters begin to decline. The reduction in *V*_oc_ is linked to enhanced non-radiative recombination at defect sites, which lowers the quasi-Fermi level splitting.^22^ Similarly, *J*_sc_ decreases as more photogenerated carriers are captured by traps before reaching the electrodes. The FF also deteriorates due to increased recombination currents and reduced carrier collection performance. Collectively, these effects result in a marked drop in PCE at higher *N*_T_ values. This behavior is consistent with the Shockley–Read–Hall (SRH) recombination mechanism, where the recombination rate increases in proportion to defect density.^23^ At high *N*_T_, trap-assisted recombination becomes dominant, shortening carrier lifetimes and diffusion lengths, which suppresses both current output and voltage.

Thus, maintaining a low defect density (≤1 × 10^14^ cm^−3^) in the BaZrSe_3_ absorber is crucial for achieving high device efficiency. Strategies such as post-deposition annealing, interface passivation, and improved material crystallinity are therefore essential to minimize *N*_T_ and preserve photovoltaic performance.

### Effect of operating temperature

**Fig. 7:**
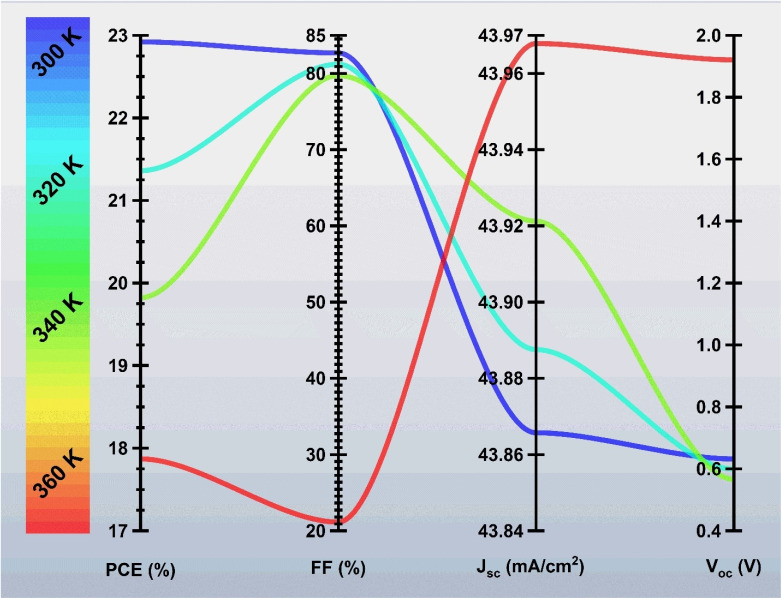
Effect of temperature range (300–360 K) on the photovoltaic performance of the device.

The working temperature has a strong influence on the photovoltaic performance of BaZrSe_3_-based solar cells. Fig. 7 shows the impact of the environment temperature on the device performance. As the environmental temperature increases from 300 K to 360 K, the device PCE drops significantly from 22.92% to 17.87%, accompanied by a drastic reduction in the FF from 82.86% to 21.15%. This degradation was explained by several temperature-induced mechanisms. First, higher temperatures increase the intrinsic carrier concentration in the absorber, which enhances recombination rates, particularly *via* Shockley–Read–Hall (SRH) and Auger processes.^24^ As a result, the *V*_oc_ decreases due to a narrowing of the quasi-Fermi level splitting. Second, elevated temperatures reduce carrier mobility by enhancing phonon scattering, which degrades charge transport and extraction efficiency. Finally, higher thermal energy was increasing the saturation *J*_0_, leading to a steeper decline in *V*_oc_ and FF.

The combined effect of these mechanisms explains the observed sharp drop in both PCE and FF with rising operating temperature. Thus, BaZrSe_3_ devices exhibit optimum performance near room temperature, while elevated operating conditions accelerate recombination and resistive losses, limiting efficiency.

### Influence of back contact material

**Fig. 8:**
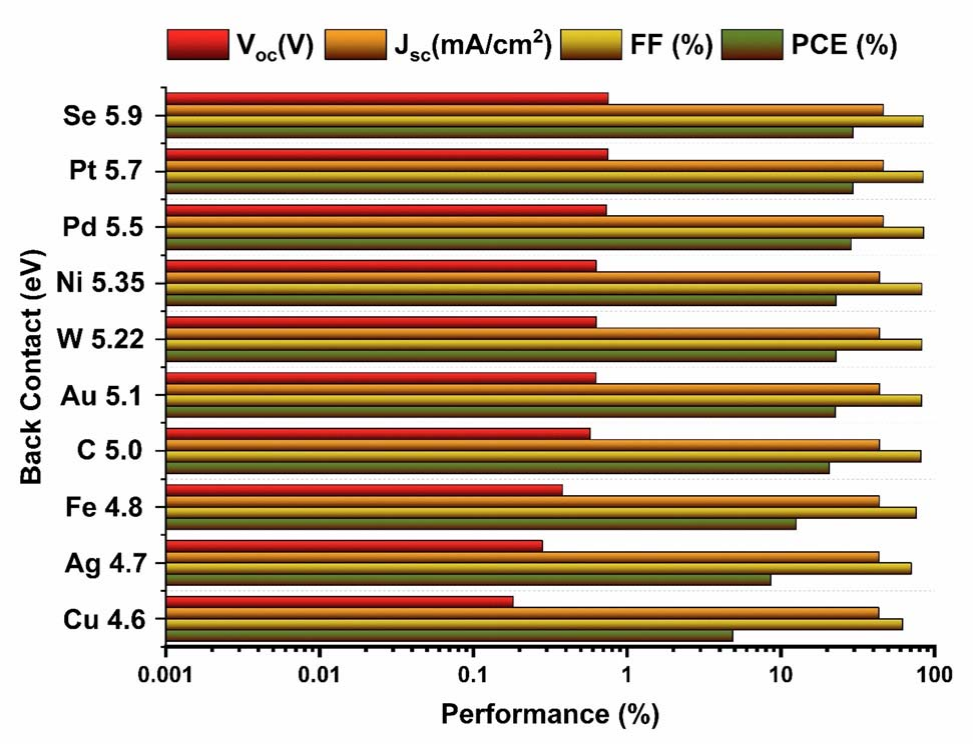
Impact of varying back metal contacts on device characteristics of the heterojunction solar cell.

Fig. 8 shows the dependence of the solar cell parameters on the choice of back metal contact, where the work function was varied from 4.6 to 5.9 eV. The back metal contact plays a crucial role in determining carrier extraction and recombination dynamics in BaZrSe_3_-based solar cells. By varying the back-contact work function from 4.6 eV to 5.9 eV, a clear trend was observed where the device performance improved significantly with increasing work function. The best performance was achieved with selenium (Se), having a work function of 5.9 eV, yielding a PCE of 29.46%, FF of 84.37%, *J*_sc_ of 46.38 mA cm^−2^, and *V*_oc_ of 0.7528 V.

This enhancement was attributed to the improved energy-level alignment between the BaZrSe_3_ absorber and the high work function back contact. A higher work function metal reduces the Schottky barrier height at the interface, ensuring better hole collection while minimizing carrier recombination at the back surface.^25^ Moreover, the formation of an ohmic-like contact enhances charge transport efficiency, leading to higher FF and *V*_oc_.

Conversely, low work function contacts create unfavorable band bending, which result in barrier formation, charge accumulation, and higher recombination losses. Thus, the observed improvement at 5.9 eV demonstrates that a proper selection of back contact material is essential for maximizing device efficiency.

### Performance comparison between initial and optimized solar cell structures

**Fig. 9:**
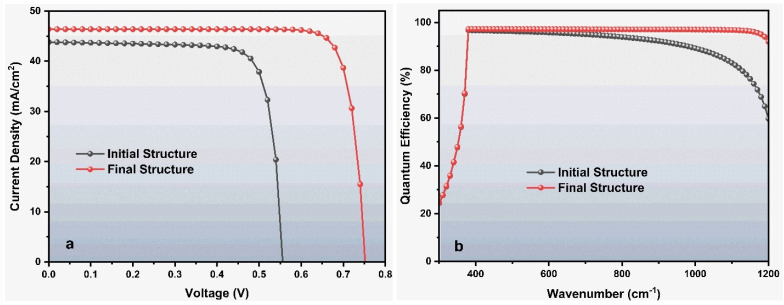
Comparative analysis of the initial *versus* optimized heterojunction solar cell.

Fig. 9 compares the *J*–*V* characteristics and QE spectra of the initial and optimized ZnO:Al/ZnO/ZnS/BaZrSe_3_/Au solar cell. A significant enhancement in device performance is observed after optimization. The PCE increases from 19.46% to 29.46%, accompanied by an improvement in the FF from 79.77% to 84.37%. The *J*_sc_ also rises from 43.80 to 46.38 mA cm^−2^, while the *V*_oc_ increases markedly from 0.5568 V to 0.7528 V. Additionally, the QE spectrum of the optimized device exhibits a noticeable enhancement across the visible region, reflecting superior photon-to-electron efficiency.

The improvement in *J*_sc_ is primarily attributed to the enhanced absorption within the BaZrSe_3_ absorber layer and optimized charge carrier transport at the ZnS buffer/absorber interface. By tailoring the thickness, doping density, and defect concentration, the absorber is able to generate a higher density of photogenerated carriers, while also minimizing recombination losses.^26^ The increased carrier lifetime and reduced series resistance facilitate more effective charge extraction, leading to a measurable rise in photocurrent density.

The notable increase in *V*_oc_ from 0.5568 V to 0.7528 V was explained by the improved band alignment and reduction in interface recombination. A higher acceptor concentration in the absorber, together with the optimized donor density in the buffer, establishes a stronger built-in electric field across the junction. This enhances charge separation and suppresses carrier recombination at defect states, thereby allowing a larger splitting of quasi-Fermi levels, which manifests as a higher *V*_oc_.

The FF improvement, from 79.77% to 84.37%, arises due to the reduction of resistive losses and suppression of recombination at both the front and back contacts. The introduction of a back contact material with a higher work function (5.9 eV) ensures a favorable ohmic contact with the p-type absorber, which minimizes barrier formation and enhances carrier extraction efficiency. Together, these factors lead to a more ideal diode behavior, reflected in the higher FF.

The PCE enhancement, from 19.46% to 29.46%, results from the cumulative effect of increased *J*_sc_, *V*_oc_, and FF. Importantly, the QE spectrum demonstrates stronger absorption and carrier collection across the solar spectrum, which confirms that the optimized device structure effectively utilizes incident photons and mitigates recombination losses.

Overall, the combined improvements in absorber thickness, doping densities, defect management, buffer optimization, and contact engineering contribute synergistically to the superior performance of the final device. This highlights the critical role of interface engineering and defect passivation in realizing high-efficiency BaZrSe_3_-based solar cells.

## Conclusion

The numerical simulation analysis was carried out on ZnO:Al/ZnO/ZnS/BaZrSe_3_/Au heterojunction solar cells to determine the important structural and electrical aspects determining performance. The results demonstrated that absorber thickness optimization, defect density control, and careful adjustment of doping levels are essential for enhancing photovoltaic characteristics. An absorber thickness of 2.0 μm, a ZnS buffer thickness of 20 nm, and optimized doping concentrations (*N*_A_ = 10^18^ cm^−3^ and *N*_D_ = 10^19^ cm^−3^) produced balanced carrier generation and transport with minimal recombination. Maintaining the defect density below 10^14^ cm^−3^ was found critical to sustaining high performance. While temperature variations adversely affected the device due to increased recombination, engineering the back metal contact with a high work function substantially improved efficiency, resulting in an optimized PCE of 29.46% with excellent *J*_sc_, *V*_oc_, and FF values. Overall, the study underscores the significance of absorber–buffer interface engineering, defect passivation, and contact optimization in achieving high-efficiency BaZrSe_3_-based solar cells. The insights gained not only validate BaZrSe_3_ as a promising lead-free perovskite-like material but also provide design strategies for further experimental development of stable, environmentally friendly thin film photovoltaic devices.

## Author contributions

Elsammani Ali Shokralla: writing – review & editing, validation, Arslan Ashfaq: writing – original draft, conceptualization, Ubaid Ur Rehman: writing – review & editing, Hind Albalawi: data curation, supervision, Zahra Bayhan: data curation, Sarah A. Alsalhi: formal analysis, validation, Shoug M. Alghamdi: resources, formal analysis, M. Musa Saad H.-E.: conceptualization.

## Conflicts of interest

There are no conflicts to declare.

## Data Availability

This study was carried out using Numerical simulator SCAPS-1D.
